# Local subcutaneous lidocaine injection for the treatment of complex regional pain syndrome: a case report and literature review

**DOI:** 10.3389/fneur.2023.1232199

**Published:** 2023-08-14

**Authors:** Yaping Su, Zhenyu Li, Qian Wang, Hui Tang

**Affiliations:** ^1^Department of Pharmacy, Shandong Provincial Hospital Affiliated to Shandong First Medical University, Jinan, China; ^2^Department of Pharmacy, The People's Hospital of Xin Tai City, Taian, China; ^3^Stem Cell Clinical Institute, Shandong Provincial Hospital Affiliated to Shandong First Medical University, Jinan, China

**Keywords:** lidocaine, local subcutaneous injection, complex regional pain syndrome, analgesic therapy, case report

## Abstract

A 14-year-old child was diagnosed with complex regional pain syndrome (CRPS) after bromhidrosis surgery. She experienced a stinging, knife-like, and intermittent attack pain, accompanied by numbness of both upper limbs and limited movements. Ultrasound-guided radiofrequency surgery on the peripheral nerve did not reduce pain. Then, gabapentin 300 mg three times a day and 2% lidocaine by local subcutaneous injection once a day for 3 days were administrated. After the local subcutaneous injection of lidocaine, the pain was significantly relieved, and the pain induced by skin touch at the scar disappeared. No pain recurred after the 1-month follow-up. An evidence-based literature review showed that local or systemic intravenous lidocaine was used to reduce adult CRPS symptoms but less has been reported in children. In our case, a local subcutaneous injection of 2% lidocaine in a child for CRPS treatment was reported to be effective in relieving complex local pain with favorable outcomes. Though further high-quality randomized controlled trials are needed to investigate the effects and safety of local subcutaneous lidocaine injection on pain relief in children with CRPS, it could still provide a relatively safe and effective adjuvant therapy for minor patients.

## 1. Introduction

Complex regional pain syndrome (CRPS) is usually secondary to primary trauma and may be a syndrome of severe intractable, polytropic pain following fracture, postoperative or unintentional minor trauma, characterized by malnutrition and dysfunction (1). The fourth edition of the Diagnosis and Treatment Guidelines for CRPS in 2013 pointed out that the main clinical features are acute pain inconsistent with the original trauma, and clinical manifestations are pain, edema, erythema, hyperthermia, hypofunction, and so on. There are two typical types of sympathetic pain disorders: CRPS type I, which has no nerve damage, and CRPS type II, which has definite nerve damage and typical neuropathic pain features (2). The incidence of CRPS was 5–25 per 1,00,000, the ratio of male to female was 1:2-3, and the ratio of upper limb to lower limb was 2:1 (3), but the pathological pathogenesis of CRPS was still unknown.

The primary treatment of CRPS is multimodal pain management, which includes bisphosphonates, glucocorticoids, non-steroidal anti-inflammatory drugs, tricyclic antidepressants, antiepileptics, NMDA receptor antagonists, and drugs such as calcitonin and Botox. In addition, there are also other options such as psychological therapy, physical therapy, surgery, and interventional therapy. Minimally invasive interventional therapy mainly includes sympathetic nerve block, spinal cord electrical stimulation, and ultrasound-guided pulsed radiofrequency. Surgical treatment mainly includes lesion of dorsal root of spinal cord and neurolytic sympathetic procedures (1, 2).

We present a unique case of a child detailing the use of local subcutaneous lidocaine injection, which is poorly documented in the literature. An evidence-based literature review was conducted to help us scientifically analyze and grasp the safety and effectiveness of analgesic drugs.

## 2. Case presentation

A 14-year-old child (168 cm, 58 kg) had been diagnosed with complex regional pain syndrome (CRPS) on 28 October 2022. Two months after a bromhidrosis surgery, the patient developed axillary pain with numbness in both upper limbs. The pain site was the axillary region, radiating to both upper limbs with pain numbness and limited movements. The numerical rating scale (NRS) was 8 points. The patient reported pain as stinging, knife-like, and intermittent attacks, with a frequency of 6–8 episodes per day, each lasting 10–20 min. The pain was worse at night than during the day, occasionally interfering with sleep. The child had no significant weight loss and denied a history of chronic medical conditions such as high blood pressure.

**Figure 1:**
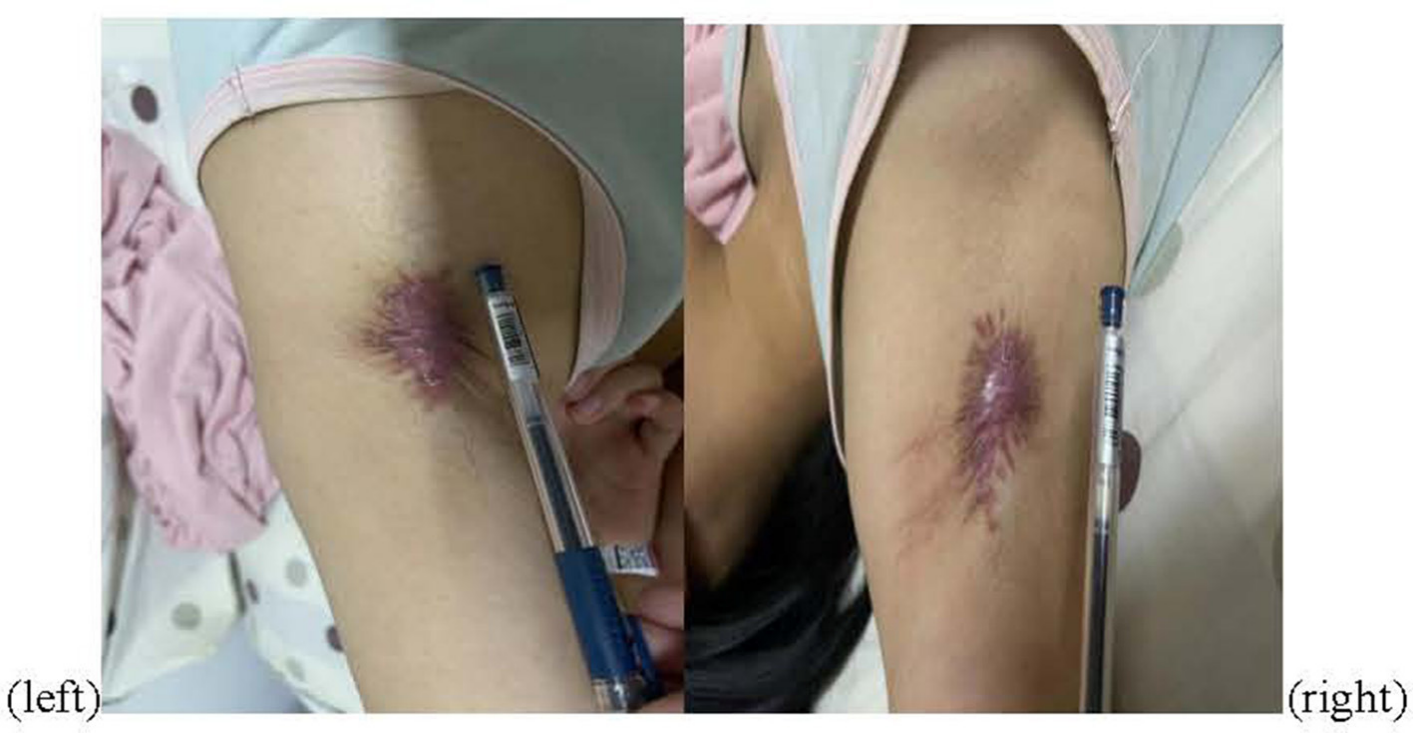
Postoperative figure.

The patient received one local injection of scar softening needle on 14 October 2022 and then applied mupirocin ointment and human epidermal growth factor gel to the scar. The color of the scar deepened and the contracture improved, but the pain did not improve significantly. Physical examination revealed bilateral axillary surgical scars of 5 ^*^ 2 cm (Figure 1), peripheral skin contracture, local tenderness (++), limited movement of both upper limbs, abductor lift of 120°, forward flexion lift of 150°, back extension test to the medial margin of scapula, random contact of both upper limbs on the ulnar side caused pain, and no atrophy of the skin, tissues, and muscles of both upper limbs. The ulnar touch induced pain in both upper limbs (+), severe pain in the left armpit, numbness in the bilateral axilla of both upper limbs, and contracture of the hands and feet during the attack. The electromyography-evoked potential report showed no significant abnormalities in nerve motor conduction, sensory conduction, F-wave, or muscles (Table 1). Bilateral axillary ultrasound, blood, urine, and stool tests were normal. The patient met the Budapest diagnostic criteria for CRPS established by Harden in 2007 (4).

**Table 1:** Electromyography/evoked potential report: the results of electromyography.

**Muscle name**	**Left/right**	**Insertion potential**	**Fibrillation potential**	**Positive sharp wave**	**Beam flutter potential**	**Myotonia**	**Amplitude**	**Polyphase wave**	**Time limit**	**Strong recruitment**	**Strong recruitment potential (mv)**
Musculi interossiae I	L	Normal	0	0	0	0	Normal	Normal	Normal	Interference phase	1.2
Radial hip flexors	L	Normal	0	0	0	0	Normal	Normal	Normal	Interference phase	1.9
Biceps	L	Normal	0	0	0	0	Normal	Normal	Normal	Interference phase	1.1
Deltoid	L	Normal	0	0	0	0	Normal	Normal	Normal	Interference phase	2.0

## 3. Therapeutic intervention and results

The patient was admitted to the hospital on 30th October and was given oral ibuprofen. Flurbiprofen ester gel ointment was applied to her right armpit for pain relief. On 1st November, radiofrequency surgery was performed on the left periaxillary nerve under ultrasound guidance, and 1 ml anti-inflammatory analgesic solution (2% lidocaine l ml +0.9% sodium chloride solution 2 ml + dexamethasone 5 mg) was given. The patient reported unbearable pain during the surgery. On the 1st day after the surgery, the patient reported increased pain in the left axilla, with no significant improvement in pain duration and attack frequency. The NRS score was 9 points. The patient was given gabapentin 300 mg, three times a day. However, after taking gabapentin, the child experienced dizziness and drowsiness. Considering that gabapentin was highly correlated with adverse reactions, the patient refused to continue taking gabapentin. After a comprehensive evaluation by clinical pharmacists and doctors, the patient was given a 3 ml local subcutaneous injection of 2% lidocaine. On 5th November, the patient reported significant relief in bilateral axillary pain, and the NRS score decreased from 8 to 3 points at the onset of pain. No adverse reactions were observed during the administration. The patient was discharged immediately. After 1 month of discharge, the patient was followed up by the clinical pharmacist, and the frequency and duration of the pain were significantly relieved. The pain disappeared completely with the subsequent application of lidocaine gel patches.

## 4. Discussion

Lidocaine was used as an adjunct to CRPS, and local subcutaneous injection was rarely published (5–9). To date and to the best of our knowledge, only eight cases of CRPS with different lidocaine administration methods were retrieved (Table 2), of which six were adults and two were children The history of CRPS was more than 1 month, and the longest duration was 7 years. The pain was mainly concentrated in the upper and lower limbs and affected the patients' normal life. Of the two children, one received a 5% lidocaine local patch (10) and the other received a local block of 0.5% lidocaine 15 mL and 0.25% bupivacaine 20 mL, followed by an intravenous infusion of 250 mg lidocaine (11). Among the six adult patients, a 26-year-old patient was given intravenous medication at 1–2mg/kg/h for 4 h (12), one patient was given 5% lidocaine ointment three times a day (13), and four patients were given a local block therapy with combination medication (11, 14–16). Except for one patient who received a systemic infusion of lidocaine and ketamine without any pain relief (17), all the other patients achieved varying degrees of pain relief, of which two patients did not achieve sustained pain relief (11, 12).

**Table 2:** Case report of different lidocaine administration modes for CRPS.

**First author**	**Patients**	**Age**	**Lidocaine administration**	**NRS**	**Pain relief**	**Duration of CRPS**	**Position and type**	**Previous treatments**	**ADR**
Rickard et al. (12)	1	26	Intravenous administration 1–2 mg/kg/h for 4 h	8	NRS decreased from 8 to 6, but did not persist and recurred 2 months later	7 years	Left upper limb was injured, affecting the contralateral function	Glucocorticoids + opioids + gabapentin 0.3 g/tid	Mild dizziness
Frost (10)	1	10	5% Lidocaine patch (12 h/d, 1 month)	(-)	Sustainable pain relief	5 years	Left lower limb CRPS I	Gabapentin 0.3 g/tid, Amitriptyline 25 mg qn, Tramadol 50 mg/q8h Baclofen 10 mg/bid, Carbamazepine 200 mg/bid, Clonazepam 0.5 mg qn In October, it was changed to Doxazosinzaleplone1 mg/d, mesylate 5 mg	(-)
Hanlan et al. (13)	1	65	5% Lidocaine ointment tid	9	Sustainable pain relief	>2 months	Traumatic cervical spinal cord injury resulting in restriction of the right hand CRPS II	Pregabalin 600 g/d, Prednisolone 50 mg, Calcitonin 200 IU	(-)
Herschkowitz and Kubias (17)	1	47	Lidocaine and ketamine infusion therapy	6–7	No relief	>6 years	Right forearm CRPS I	Regional block + opioids + gabapentin 0.3 g/tid	(-)
Patel and Aiello (14)	1	65	0.5 ml 1% lidocaine and 20 mg dexamethasone were injected into the flexor tendon sheath	(-) Acute pain	Sustainable pain relief	>3 years	Right hand right forearm CRPS I	Multimodal therapy: occupational and desensitization therapy; Medication: nortriptyline 60 mg Oxycodone acetaminophen 5/325 mg Duloxetine 60 mg/bid	Not found
Wang et al. (15)	1	32	Betamethasone + 0.5% lidocaine was injected locally into the right carpal tunnel and pain point	10	Complete pain relief, NRS decreased to 1	>1 month	Right wrist and upper limb CRPS I	Multimodal therapy: occupational and desensitization therapy; Medication: oral corticosteroid, opioid agonist, and antidepressant	Not found
Toda et al. (16)	1	35	10 regional blocks of 1% lidocaine at 0.5 mL/kg were administered in the dorsal vein of the wrist	10	Complete pain relief	>1 years	Right hand CRPS I	Transcutaneous electrical nerve stimulation, occupational therapy and carbamazepine	Mild dizziness
Maneksha et al. (11)	1	12	15 mL 0.5% lidocaine + 20 mL 0.25% Bupivacaine+ 250 mg lidocaine iv	10	Mild pain relief, NRS decreased to 7	>5 months	Right foot and lower limb CRPS I	Physical therapy, regional nerve block and medication: Tramadol 50 mg Gabapentin 0.3 g/tid Amitriptyline 10 mg	Mild toxic symptoms with perioral tingling and some slurred speech occurred during intravenous administration

NRS, numerical rating scale; tid, three times a day; bid, twice a day; iv, intravenous injection; ADR, adverse drug reaction.

In addition, we retrieved seven clinical trials of lidocaine for CRPS (Table 3), including three observational studies and four prospective studies. Intravenous lidocaine was administered in two clinical trials, one sustained subcutaneous infusion of lidocaine 3–5μg/ml (18), and one intravenous infusion of lidocaine 5mg/L for 5 days (19). There were three trials of lidocaine combined with other agents for regional block (5, 7, 8), and one lidocaine patch as an additional analgesic (6). With the exception of 49 patients, mentioned in a study by Schwartzman et al. (19), who relapsed after 6 months of pain relief, all patients in these trials achieved significant pain relief and no recurrence. Moreover, the duration of remission was not associated with mean lidocaine levels or highest measured lidocaine levels but was significantly associated with baseline pain intensity. There was a randomized controlled double-blind trial of lidocaine for regional block. Taskaynatan et al. found that in 22 patients with CRPS, 2% lidocaine combined with 40 mg methylprednisolone did not provide lasting pain relief and only relieved pain for 1 h (9).

**Table 3:** Clinical trials of different lidocaine administration modes for CRPS.

**First author**	**Patients**	**Age**	**Lidocaine administration**	**NRS (VAS)**	**Pain relief**	**Duration of CRPS**	**Position and type**	**Previous treatments**	**ADR**
Linchitz and Raheb (18); observational study	5	37–63	Serum concentration is maintained by continuous subcutaneous infusion with infusion pump for 3-5μg/mL	7–10	Sustainable pain relief	3–8 years	(-)	Conventional treatment is invalid (standard medical therapy and physical therapy)	(-)
Schwartzman et al. (19); observational study	49	18–62	Intravenous administration was titrated slowly to 5 mg/L for 5 days	5–10	NRS decreased to 3.4 after 1 month and slightly decreased after 6 months	0.5–25 years	Upper and lower limbs and spread to the chest, back, and feet	Conventional treatment is invalid (standard medical therapy and physical therapy)	No serious complications were found. Mild side effects include nausea, fatigue, bradycardia, tachycardia, and atrial arrhythmia
Varitimidis et al. (5); observational study	168	19–78	25 ml 0.5% lidocaine +125 mg methylprednisolone for hand venous regional block	NRS >4, 81 patients; NRS 6.5–10	148 patients reported only mild pain (0–2) and 20 reported pain relief	>1 month	Double upper limbs CRPS I	Conventional treatment is invalid (standard medical therapy and physical therapy)	Not found
Calderón et al. (6); single-center, prospective, observational study	10	28–91	5% lidocaine ointment for additional analgesia	7.9	Sustainable pain relief, NRS decreased to 3.9.	>6 months	(-)	Anticonvulsants, antidepressants, non-steroidal anti-inflammatory drugs, opioids, and nerve blocks and radiofrequency	Local skin reactions (itching, redness, or dry skin)
Singh et al. (7); single-center, prospective, observational study	33	48–62	Regional block: Shoulder joint: 40 mg methylprednisolone +5 ml 2% lidocaine; radial, median, and ulnar nerve: 10 mg methylprednisolone + 1.5 ml of 2% lidocaine	7–10 (VAS)	Sustainable pain relief, VAS decreased to 2.7.	>1 month < 1years	Double upper limbs	No other treatment	(-)
Paraskevas et al. (8); single-center, prospective study	17	33–72	Dorsal venous block of hand 15 mg guanethidine +1 mg/kg lidocaine twice a week for 5 weeks; 10 mg guanethidine +1 mg/kg lidocaine, once every 2 days, 20 times	8–10 (VAS)	Sustainable pain relief	5–67 months	Left and right hand	NSAIDs + opioids + calcitonin injection and physical therapy	(-)
Taskaynatan et al. (9); prospective, randomized, placebo study	22	20–25	40 mg methylprednisolone +10 ml 2% lidocaine for cubital regional block three times	>5	Relieved 1 h later, but no significant difference between 1 week and 1.5 months later	>3 months	Unilateral upper limb	(-)	Nausea, dizziness, tinnitus, flushing, and pruritus. All symptoms resolved spontaneously in a few hours

NRS(VAS), numerical rating scale (visual analog scale); tid, three times a day; bid, twice a day; ADR, adverse drug reaction; NSAIDs, non-steroidal anti-inflammatory drugs.

In terms of safety, we found that high concentrations of systemic lidocaine were more likely to cause adverse effects, including nausea, dizziness, fatigue, bradycardia, tachycardia, and atrial arrhythmias (Table 3) (12, 19). The adverse reactions in patients using lidocaine patches were mild, mainly skin reactions, which could be recovered after drug withdrawal (6). Patients who received lidocaine regional block had few adverse reactions, mainly mild dizziness (9, 11, 16).

In our case, the patient was a 14-year-old child who developed CRPS in both arms after surgery with increased pain following peripheral nerve radiofrequency therapy similar to the case reported by Frost et al. in (10). The patient experienced significant adverse reactions after taking gabapentin. The pain was significantly relieved 3 days after a local subcutaneous injection of 2% lidocaine, and no adverse reactions were observed during the administration.

Based on the above findings, a 5% lidocaine patch appears to have a significant analgesic effect on CRPS. Intravenous lidocaine could provide temporary relief, but it does not last long term. The usual dose of lidocaine for local block is 0.5–2%, and the pain relief is obvious, which is similar to the case we reported. It is suggested that topical lidocaine might relieve the pain of CRPS better and has a favorable safety. However, these clinical trials have been conducted on adults, and there have been few studies on adolescents.

By reviewing the literature, we attempted to analyze and speculate the reasons why local subcutaneous injection of lidocaine could relieve the pain of CRPS. A large number of ion channels related to pain regulation, such as voltage-gated sodium channel, potassium channel, and calcium channel, are distributed in the central and peripheral nervous system, which are closely related to pain perception (20, 21). Lidocaine is a selective Na+ channel blocker with strong membrane stabilization. It can selectively block the inward flow of Na+, block the K+ channel, prevent depolarization of damaged and dysfunctional nerves that are implicated in chronic pain (20, 21), increase the cellular efflux of glutamic acid and potassium, decrease excitability, and diminish neuronal transmission of pain signals, achieving the purpose of pain treatment (21, 22). Subcutaneous injection of lidocaine creates a high drug content in the spinal ganglia through the intradermal nerve terminal receptor pathway, severing the ring of pain stimulation, and thereby relieving pain (23).

In summary, we reported a case of a child treated with a local subcutaneous injection of 2% lidocaine for CRPS pain. At present, there is still no specific treatment for juvenile CRPS at home and abroad. There are also few clinical studies and systematic analyses on CRPS. Through a review of evidence-based literature, we concluded that local administration or local block used to treat CRPS pain in adults provided more pain relief than systemic administration of lidocaine. Our case and review of the literature suggested that local subcutaneous injection of lidocaine might be a convenient and effective treatment for CRPS in children. However, due to the small number of clinical cases collected and the lack of rigorous randomized controlled trials, the safety of CRPS in the treatment of adolescents remains uncertain. Therefore, further high-quality randomized controlled studies are needed to investigate the effect of local subcutaneous injection of lidocaine on pain relief in children with CRPS.

## Data availability statement

The original contributions presented in the study are included in the article/supplementary material, further inquiries can be directed to the corresponding authors.

## Ethics statement

The studies involving humans were approved by Shandong Provincial Hospital Ethics Committee. The studies were conducted in accordance with the local legislation and institutional requirements. The participants provided their written informed consent to participate in this study. Written informed consent was obtained from the individual(s), and minor(s)' legal guardian/next of kin, for the publication of any potentially identifiable images or data included in this article.

## Author contributions

QW and HT contributed to the conception and design of the study and proofread all drafts. YS wrote the first draft of the manuscript. ZL wrote sections of the manuscript. HT provided guidance on the article. All authors contributed to the revision of the article, read, and approved the submitted version.
